# Providing nursing care to Ebola virus disease patients: China Ebola Treatment Unit experience

**DOI:** 10.7189/jogh.05.020301

**Published:** 2015-12

**Authors:** Jie Cao, Lingjuan Zhang, Huijun Xi, Xiaoying Lu, Danfeng Chu, Minghui Xie, Li Li, Jue Chen

**Affiliations:** 1 Urology Department of Changhai Hospital affiliated to the Second Military Medical University, Shanghai, Peoples’ Republic of China; 2 Nursing Department of Changhai Hospital affiliated to the Second Military Medical University, Shanghai, Peoples’ Republic of China; 3 Neurosurgical Department of Changhai Hospital affiliated to the Second Military Medical University, Shanghai, Peoples’ Republic of China; 4 Radiology Department of Changhai Hospital affiliated to the Second Military Medical University, Shanghai, Peoples’ Republic of China; 5 Nursing Department of Eastern Hepatobiliary Surgery Hospital affiliated to the Second Military Medical University, Shanghai, Peoples’ Republic of China; 6 Nursing Clinics of Changhai Hospital affiliated to the Second Military Medical University, Shanghai, Peoples’ Republic of China; * Joint first authorship

Since the outbreak of Ebola Virus Disease (EVD) in mid 2014, many countries and WHO sent physicians and nurses to sub–Saharan Africa. The Chinese government sent three groups of medical staff to Liberia over the period from 14 November 2014 to 15 May 2015 and built an EVD treatment unit located in the Samuel Kanyon Doe (SKD) sports stadium of Paynesville which then received patients from across the country. EVD is a highly contagious and lethal disease [1] with no effective vaccine or specific treatment regimen. Since the beginning of 2014, Ebola virus has infected more than 20 000 people and killed more than 7000 patients. WHO reported that infected medical staff are one of the groups with the highest case fatality ratios. The President of People’s Republic of China issued an order to “cure EVD patients, as many as possible, while keeping zero infection of medical staff.” With this goal, the Ministry of Health organized a panel of infectious disease prevention and treatment experts to design the Ebola Treatment Unit (ETU) in Paynesville, Liberia, and sent 3 medical teams (approximately 700 physicians and nurses) to Sierra Leone and Liberia.

The Ministry of Health designed a curriculum for ETU staff, which included: geographic and cultural orientation to West Africa, the peace keeping tasks of United Nation in Africa, contagious disease prevention and treatment in tropical zones and the use of personal protective equipment (PPE). Staff were trained in PPE dressing and undressing in simulation training centers in military medical universities and general hospitals in different regions. Regularly rehearsed patient scenarios in high ambient temperatures were an essential element of the training to ensure staff experienced some of the real life challenges of patient care in these settings.

Physicians and nurses in China in the ETU had prior training and at least 5 years of experience in epidemiology and infectious disease, respiratory, anesthesiology, general surgery and clinical nursing. Most of them were directors and head nurses in tertiary hospitals affiliated to Military Medical Universities or General Hospitals throughout China. The physician nurse ratio was 1:1.5 and 79% of nurses had more than 10 years of experience. Their age ranged from 24 to 58 years and most of them had participated in medical rescue missions following earthquake or flood disasters or had experience in sports injury treatment at large international events such as the Olympic and World Expo Games.

**Figure 1:**
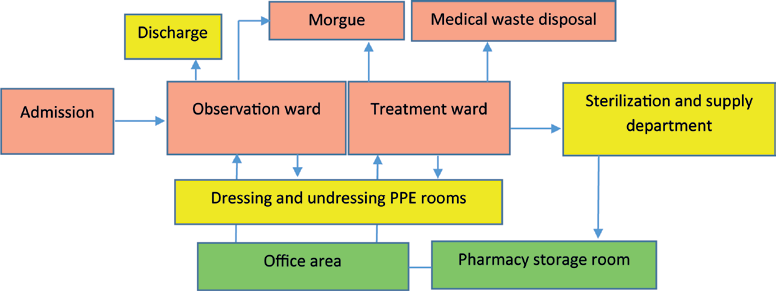
Structure map of the Ebola Treatment Unit (ETU).

The China ETU comprised an outpatient clinic, observation ward, treatment ward, infection control and an anti–epidemic department. A group of supervisors were responsible for the surveillance of infection control quality. **Figure 1** is a simplified diagram of the layout of the contaminated (in red color), semi–contaminated (in yellow color) and uncontaminated (in green color) areas of the ETU.

**Figure 2:**
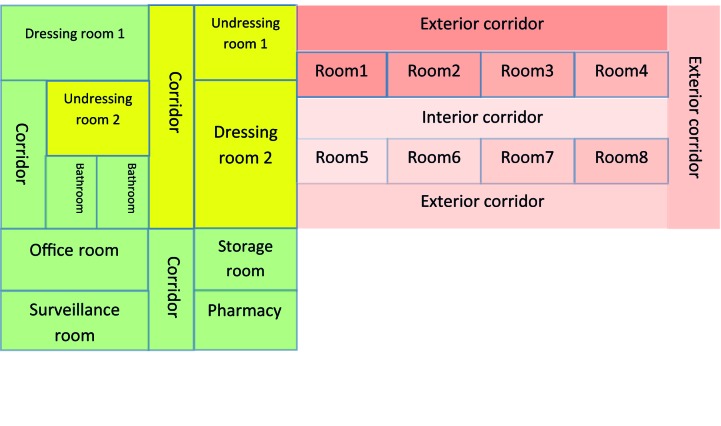
Architectural structure of observation or treatment ward in the Ebola Treatment Unit (ETU).

Within the wards of observation and treatment, there were strictly designed entering and exiting routes in order to avoid cross infection (**Figure 2**). This was arranged as a one–way system. Patients were only allowed to have daily activities in the patient’s room and exterior corridor and not allowed to enter the inner corridors which were staff–only areas. The area in red was considered as a contaminated area (colour intensity proportion to the hypothetical virus density). All the items of PPE were disposed of in dressing room 1 and dressing room 2 except water–proof boots in dressing room 1. Used boots were collected by sterilization and supply nurses after disinfecting them with 5000 mg/L chlorine solution, drying with sunshine, checking leakage with a flashlight in the dark. They were then sent to dressing room 1 as required.

By the end of 2014, researchers, caregivers and the support team at the ETU went through the “lived experience” of preparing and caring for patients with exposure as well as confirmed cases. Appropriate PPE dressing and undressing protocols were developed:

**Dressing room 1:** Wearing of split gowns with disposable socks, latex gloves, N_95_ mask, head cloth, goggle, conjunctive protective gown, suitable butyronitrile gloves, surgical cap, and waterproof boots sequentially (checking correct size by making extended motions);

**Dressing room 2:** Hand washing, taking on disposable surgical gown, facial splash shield, latex gloves, boot covers, and before entering the patient’s room wearing the outermost layer’s gloves;

**Undressing room 1:** Hand washing, taking off and then putting on gloves; splashing chlorine disinfection solution from head to feet; washing hands and taking off the disposable surgical gowns; taking off boots covers, gloves, facial splash shield, cap, and washing hands; entering the chlorine solution pool to disinfect boots for 2 minutes;

**Undressing room 2:** Opening the door with paper tissues; entering the dry pool to take off boots, using an instrument to transfer boots into recycle bucket, changing slippers; washing hands and taking off protective gown; washing hands and taking off butyronitrile gloves; taking off goggle and head cloth; washing hands and taking off gloves; taking off mask with naked hands and then washing hands and entering the shower room.

From 15 December 2014 to 20 March 2015, there were 296 patients screened at the outpatient clinics, 56 of whom were admitted to the observation ward and 11 diagnosed with EVD by Ebola PCR laboratory examination. The average age of confirmed cases was 42.3 years old; 7 were male and 4 were female. According to the common symptoms listed by Dr Jarrett [2], 100% had the symptom of weakness, 90.9% had fever >38.6°C, 81.8% had diarrhea, 72.7% had vomiting, 72.7% had muscle or joint pains, 63.6% had abdominal pain, 54.5% had general malaise or asthenia, 54.5% had anorexia, and 36.4% had unexplained hemorrhage. Four confirmed patients died while 7 recovered with treatment which included correction of low blood volume and electrolyte balance, nutritional support, pain relief, hemostasis, antipyretic treatment and management of diarrhea. Signs of severe bleeding, for example, black stools or hemoptysis, were associated with poor prognosis.

There were three shifts for nursing personnel every day, morning shift (8AM–2PM), afternoon shift (2PM–8PM), night shift (8PM–8AM) staffed by 4, 4 and 2 nurses on duty respectively. The nurse scheduling was in accordance with the “morning–morning–afternoon–night” alternation rotation mode. Nursing administrators established the nurses’ job descriptions and responsibilities. The workflow information for each shift was pasted on the office wall for ease of reference by staff.

**Figure:**
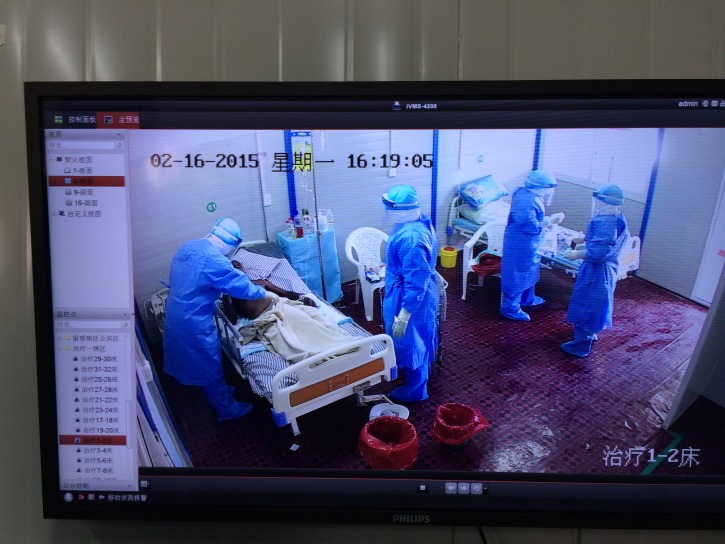
Photo: Nurse Chu Danfeng, Feng Li and Chen Dan, accompanying to Medical director Li Fuxiang of China ETU, are treating and caring for Ebola patient in the ward. (Courtesy of Cao Jie)

Nurses were responsible for: replenishing materials, medications, and disposable supplies; transcribing and processing the orders, charging nursing documents and ward diary; registering new patients; and assisting personnel to wear PPEs. When nurses entered the ward they needed to take responsibility for applying nursing rounds, taking vital signs and reporting through a calling and answering system; oral medication administration, peripheral vein catheter insertion and intravenous infusion; psychological counseling or reassurance; and supervising hygienists. In view of the particular working environment of ETU, nursing managers used job memoranda before entering the ward, cycled alternately between observation and treatment wards, and established an innovative scheduling information release system to improve the care quality and guarantee patients’ safety.

It is important to gain experience from ETUs set up during this epidemic in Africa, especially in Liberia, Guinea and Serra Leone where the health care system is particularly inadequate for appropriately managing and containing infectious diseases [3]. The correct level of isolation for patients with EVD in the ETU and which are the most essential medical and nursing interventions are still issues which are uncertain and under discussion [4]. A specialist committee is essential to provide a source of reference and advice for decision making in crucial procedures as there is little known of this deadly disease. There has been limited published experience from health care facilities exposed to the risks of transmission during invasive procedures and close body contact [5]. In our experience medical workers who had direct contact with blood and body fluid directly had more serious symptoms and signs than patients exposed through attendance at funeral ceremonies.

There have been many personal protection protocols announced by the WHO. In our experience the Principle of “Extreme Caution” is never to be underestimated in order to reach the “Zero Infection” goal among medical and nursing staff. As an emerging infectious disease, EVD is not “horrible monsters” if medical and nursing staff strictly follow the personal protection principles.
